# Dominant Temporo-Basal Glioblastoma with Rapid Progressive Aphasia: Venous-Anchored Maximal Safe Resection and Quantified Language Recovery

**DOI:** 10.3390/diagnostics16071057

**Published:** 2026-04-01

**Authors:** Valentin Titus Grigorean, Adrian Vasile Dumitru, Nicolaie Dobrin, Matei Șerban, Răzvan-Adrian Covache-Busuioc, Corneliu Toader, Andrei Marin, Carmen Giuglea

**Affiliations:** 1 Faculty of General Medicine, “Carol Davila” University of Medicine and Pharmacy, 050474 Bucharest, Romania; 2 Department of General Surgery, “Carol Davila” University of Medicine and Pharmacy, 050474 Bucharest, Romania; 3 Department of Pathology, Faculty of Medicine, “Carol Davila” University of Medicine and Pharmacy, 030167 Bucharest, Romania; 4 “Nicolae Oblu” Clinical Hospital, 700309 Iasi, Romania; 5 Puls Med Association, 051885 Bucharest, Romania; 6 Department of Neurosurgery, “Carol Davila” University of Medicine and Pharmacy, 050474 Bucharest, Romania; 7 Department of Vascular Neurosurgery, National Institute of Neurology and Neurovascular Diseases, 077160 Bucharest, Romania

**Keywords:** glioblastoma, dominant temporal lobe, aphasia, language networks, venous preservation, basal vein of Rosenthal, maximal safe resection, intraoperative neuromonitoring, cystic tumor, functional recovery

## Abstract

**Background and Clinical Significance**: Modern neuro-oncologists encounter a major challenge when dealing with glioblastomas located in the dominant hemisphere’s temporo-basal area, because their invasive nature disrupts the proximity to eloquent cortical areas (language and speech), as well as skull base venous structures, which can lead to a quick decline in function from the disruptions in these networks and the disconnection of corridor-level pathways. This manuscript illustrates the application of metric-based phenotyping, anatomically defined imaging, and venous-anchored microsurgical techniques that can aid in preserving the remaining functional reserve in patients with dominant hemisphere glioblastomas and demonstrate measurable outcomes through longitudinal follow-up data. **Case Presentation**: A 48-year-old right-handed male patient presented with a four-week history of progressively worsening symptoms consistent with a dominant hemisphere syndrome, resulting in a significant decrease in his independence (mRS 0 → 4; BI 55/100; IADL 2/8). His symptoms included non-fluent expressive aphasia with a marked inability to generate words and respond to verbal cues (BNT 8/30; SF 4 WPM). Additionally, he experienced prolonged lateralizing hemisensory decompensation and corticospinal tract dysfunction. Imaging studies revealed a large multiloculated cystic lesion located in the left temporo-basal region. The lesion displayed a thick irregular peripheral enhancement pattern with mural nodules and septa, and surrounding T2 hyperintensity extending into the temporal associative white matter, indicating disruption of the lexical–semantic networks and corridor-level tracts. Utilizing continuous SSEPS/MEPs during surgery, a skull base parallel ventral temporal corridor was developed to allow decompression of the cyst first, followed by cyst evacuation, inside-out cytoreduction, subpial dissection, and specific preservation of both superficial and deep temporal veins using selective capsular preservation at venous interface locations where necessary. Postoperative CT scans performed on POD #3 and POD #7 indicated stable decompression without hemorrhage or hydrocephalus complications, followed by rapid quantitative improvement in NIHSS (8 → 2), MoCA (18 → 26), BNT (8 → 26), SF (4 → 12), mRS (2 at discharge, 1 at follow-up), BI (85 at discharge, 95 at follow-up), and IADL (6/8 at discharge, 8/8 at follow-up). Histopathological examination confirmed a diagnosis of glioblastoma. **Conclusions**: This case study supports a model of a network- and vein-constrained glioblastoma of the dominant hemisphere in the temporo-basal region that can result in substantial restoration of language capabilities and preservation of functional reserves for additional therapies using venous-anchored subpial microsurgical approaches. The use of objective and quantifiable measures of phenotyping and longitudinal follow-up tracking could provide a reproducible method for measuring the degree of recovery of the affected network(s) and establishing safe boundaries for temporal glioma surgery.

## 1. Introduction

Temporally dominant glioblastomas represent a significant clinical concern due to their high rate of biological aggressiveness and potential to rapidly impair both language function and overall functional status as a result of disease progression and treatment [1]. The rate of neurological decline in patients with temporally dominant tumors can be quite rapid (a matter of weeks) due to several interconnected mechanisms, including tumor invasion into surrounding brain tissue, peritumoral edema, seizure activity, and localized vascular disturbance [2]. These mechanisms can impact distributed neural networks and may not only impact function in the area directly affected by the tumor; thus, even small increases in tumor size or the degree of peritumoral dysfunction may result in disproportionately large effects on language function in the dominant hemisphere. As a result, there may be limited opportunity for maximal safe resection and early administration of adjunctive therapies. Increasingly, it appears that glioblastoma should be viewed as a process that not only destroys local brain tissue but also impacts functional neural networks through mechanisms such as tract-mediated invasion, disruption of normal synaptic signaling, and modification of the excitability of the peritumoral microenvironment [3]. Recent experimental data suggest bidirectional communication between neurons and glioma cells; thus, neuronal activity may affect the growth of tumors, whereas tumor-related processes can affect neural circuit function [4].

Many clinical manifestations of glioblastoma represent either potentially reversible dysfunction due to factors such as edema, seizures, or transient disruption of signal flow versus irreversible damage resulting from structural disconnections in the central nervous system. One of the most sensitive manifestations of network vulnerability in the dominant hemisphere is progressive aphasia. Language production and lexical retrieval depend upon widely distributed networks of neurons, including the inferior temporal and temporo-basal cortex, ventral stream pathways that support semantic processing, and temporo-frontal pathways that support speech production and executive selection processes [5,6]. Thus, tumors impacting these areas may produce language syndromes that mimic those seen in vascular or neurodegenerative disorders, despite being caused by entirely different biological mechanisms [7].

Surgery for lesions in the ventral and basal temporal lobe is particularly challenging in terms of balancing the proximity of vital language networks, limbic structures, the skull base, and the variability in venous drainage. Surgical safety in this region does not solely depend on knowledge of cortical and subcortical functional anatomy; preservation of critical venous pathways is equally important. Damage to basal temporal or uncal venous drainage may result in delayed venous congestion, hemorrhagic venous infarction, and subsequent neurological decline; all of which pose significant risks to language function in the dominant hemisphere [8]. Maximal safe resection of dominant temporo-basal glioblastoma may be considered a procedure with limitations imposed by both functional networks and venous anatomy. Surgery for glioblastoma in this region typically involves decompression to reduce pressure in the operative corridor, subpial dissection to preserve the pia–arachnoid interface, and preservation of tumor capsule adjacent to critical venous junctions if a safe cleavage plane cannot be identified [9]. Parallel with these efforts, increasing importance is placed on standardized neurological and language assessments to objectively evaluate postoperative recovery and provide guidance for postoperative care [10].

We report the case of a patient who underwent a microsurgical approach to treat a glioblastoma in the dominant temporo-basal lobe that emphasized the preservation of venous drainage and functional pathways. Quantitative assessments of neurological and language recovery were obtained at serial intervals postoperatively. While individual case reports cannot serve as guidelines for management, detailed analysis of the clinical–radiological–surgical correlation may provide insights into the development of function-preserving strategies for tumors located in eloquent brain regions.

This is far more than a simple application of previously established microsurgical techniques and serves as an example of an uncommon and underrepresented surgical condition. In addition to anatomical limitations, the potential for tumor resection is further constrained by oncologic urgency, the vulnerability of high-functioning language networks in the dominant hemisphere, and dependence on critical venous drainage. The value of this case lies in its ability to illustrate how, in dominant temporo-basal glioblastomas, the ultimate limits of tumor resection may be determined by a common threshold that defines neurofunctional (language) and vascular (basal venous drainage) boundaries, beyond which compromise can result in irreparable damage. Thus, while the decision to preserve venous structures was primarily made to protect against postoperative complications, it also played an important role in defining the degree to which the patient’s neurofunction could be preserved during surgery. The evolution of the surgical strategy from maximal tumor removal to network-preserving cytoreduction further illustrates how the patient’s rapid preoperative decline in speech, the technical difficulty of separating the glioblastoma from adjacent neural tissue, and the serial quantification of postoperative recovery together provide a framework for assessing when a glioblastoma in an eloquent area is not simply difficult to remove surgically but also biologically aggressive and functionally unstable, thereby warranting a more individualized operative approach.

## 2. Case Presentation

The data from this case study are based on a 48-year-old right-handed male who suffered from a progressive deterioration in his neurological and language abilities over a four-week period. At the time of admission, the patient could barely speak in full sentences and had total loss of independence (modified Rankin Scale 0 → 4). It appears highly unlikely that this level of language function loss occurred as a result of purely gradual atrophy in the dominant hemisphere over the course of a few weeks. Therefore, there appears to have been some degree of disruption to the functioning of networks in the dominant hemisphere, resulting in rapid changes in language ability.

A comprehensive evaluation of the patient’s language skills was completed. Results showed that the patient had a form of non-fluent aphasia characterized by severe anomia, as shown by performance on the Boston Naming Test (8/30), and severely decreased semantic fluency (four words/minute). The patient’s articulation was preserved; therefore, the patient’s language deficits appeared to be due to disruptions in higher-order language processes rather than articulatory-motor processes. Additionally, the patient was significantly impaired on sentence repetition tasks and did not improve when cued, providing additional support that the lesion disrupted the dominant temporo–perisylvian network and negatively impacted lexical–semantic access. The deficits seen in this patient were not characteristic of a focal cortical syndrome; instead, they demonstrated disruption in the functioning of distributed language circuitry. Therefore, the patient’s deficits suggest that the tumor has extended beyond localized damage to network-level destabilization. Thus, the significance of this case does not lie in the presence of aphasia, but in the nature of the aphasia. The rapid development of deficits in all areas of language suggest a lesion that disrupts both cortical “hubs” and their subcortical connections. All evaluations were done using standardized language assessment tools in the patient’s native language.

About two weeks before the patient’s admission, the patient experienced a single 24 h episode of transient right-sided paresthesias, as well as a continued decline in aphasia. The clinical significance of this episode is found in its indication that the process responsible for these symptoms was dynamic and capable of impacting either contiguous or functionally related systems. Possible mechanisms for this episode include focal epileptiform discharges, perilesional cortical irritation, edema-induced conduction block, or transient network transmission anomalies secondary to developing mass effects. Of greater importance, the concurrent occurrence of transient symptoms and continued language decline raise the possibility of a pre-disconnection phase, where temporarily functional but vulnerable eloquent networks continue to be activated periodically before becoming permanently impaired. Transitional states like those described above may be underdiagnosed in patients with glioblastomas and represent an important opportunity for surgery to optimize potential recovery.

Upon admission, the patient was fully awake (Glasgow Coma Scale = 15); however, the overall neurological deficit (NIH Stroke Scale = 8) was considerable, with most of the deficit being caused by language deficits. Cognitive evaluation (Montreal Cognitive Assessment = 18/30) showed specific deficits in language function, while orientation and visuospatial function were normal. These findings provided evidence that the deficit was due to a focal process that impairs network function rather than general cognitive decline. Motor examination revealed mild upper motor neuron signs in the right arm (Medical Research Council grade 4+/5), hyperreflexia, and an extensor plantar response, without evidence of neglect or sensory extinction. The significant language dysfunction, combined with normal global awareness and cognition, indicated that the decline in function was not solely due to tumor burden but rather a selective collapse of the functional organization of the dominant hemisphere.

Collectively, the clinical findings provide evidence for the hypothesis that the patient had a dominant temporo-basal lesion with subcortical spread affecting language networks and descending motor pathways. Although the differential diagnosis included primary brain tumors, metastatic disease, demyelinating diseases, infections, and subacute vascular events, the distinguishing feature of this case was the disparity between the rate and extent of functional decline and the patient’s well-preserved neurological status at admission. This disparity indicates a glioblastoma that can induce early functional network dysfunction before extensive structural damage occurs clinically. From a surgical perspective, this has implications for the surgeon: the surgeon is tasked not only with removing a mass from eloquent cortex but also intervening within a small window of time, where the functional substrate has become significantly destabilized but not irreversibly damaged.

Imaging Findings.

**Figure 1:**
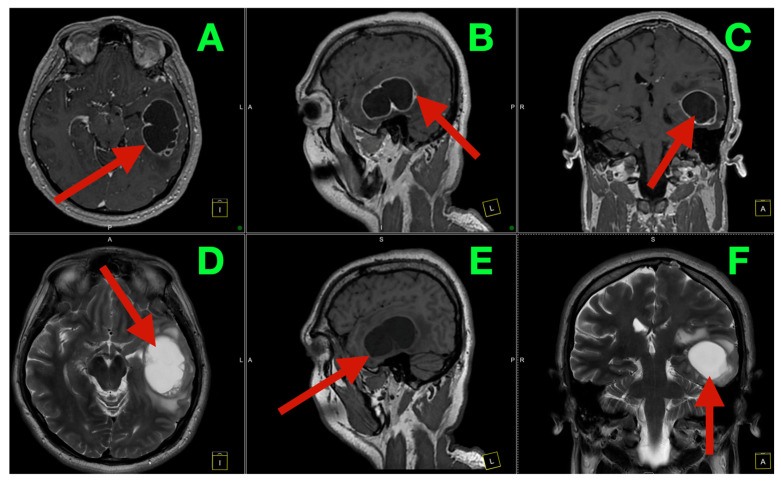
Preoperative MRI of the dominant left temporo-basal cystic tumor. (**A**) Axial T1 post-contrast: large left temporo-basal multiloculated cystic mass (arrow) with thick, irregular rim enhancement, enhancing septations, and focal mural nodularity, deforming the basal temporal cortical ribbon (inferior temporal gyrus/temporal pole region), concordant with progressive expressive aphasia and cue-resistant anomia. (**B**) Sagittal T1 post-contrast: skull base–adjacent footprint (arrow) along the middle cranial fossa floor with inferior temporal mass effect. (**C**) Coronal T1 post-contrast: enhancing mural interfaces (arrow) near the perisylvian corridor/Sylvian fissure, topographically aligned with dominant temporo-perisylvian language output circuitry. (**D**) Axial T2: hyperintense cystic compartments (arrow) with perifocal T2 hyperintensity tracking into temporo-subcortical associative white matter. (**E**) Sagittal T1 non-contrast: volumetric cystic component (arrow) defining the inferior temporal surgical corridor toward the skull base. (**F**) Coronal T2: surrounding parenchymal signal abnormality extending toward the insular–Sylvian region along the expected course of MCA M2–M3 opercular branches, supporting network-level disruption beyond the cyst wall.

His preoperative MRI demonstrated a large multi-cystic mass located in the left temporo-basal region (Figure 1A–F). Contrast-enhanced T1-weighted images of the lesion showed a thick, irregular rim of enhancement with internal septations and mural nodule formation, consistent with a cystic-solid neoplasm. There was a significant amount of surrounding T2 signal hyperintensity extending into the adjacent temporal white matter, consistent with edema and disruption of the associated fiber tracts. The lesion was located in the inferior and basal temporal regions, which are consistent with the patient’s progressive expressive aphasia and naming impairments.

The fluid-sensitive sequences showed a large area of T2 signal hyperintensity surrounding the cystic components of the lesion, consistent with edema and involvement of the temporo-subcortical white matter fibers. The location of the tumor near the temporo-perisylvian language circuit and the nearby projection fibers provides a plausible anatomical basis for the patient’s symptoms, consisting of both aphasia and upper motor neuron signs.

Preoperative surgical planning

General anesthesia was used during the surgery, along with continuous somatosensory-evoked potential and motor-evoked potential monitoring. The primary goal of preoperative surgical planning was to safely remove the cystic portion of the tumor while preserving the venous drainage pathways of the temporo-basal region. Special emphasis was given to preserving the patency of the basal temporal veins, as these were considered a critical constraint to safe tumor removal.

Following administration of dexamethasone and osmotherapy, the patient’s head was secured in a Mayfield skull restraint system and positioned for a subtemporal approach to access the cystic component of the tumor. A skin incision was made immediately posterior to the superficial temporal artery, and a craniotomy approximately 3.5 × 4 cm was performed at the junction of the anterior zygoma and the temporal squama. The inferior margin of the craniotomy was drilled to conform to the middle cranial fossa floor, and the superior temporal line was undercut to create a retractor-free subtemporal corridor.

Intraoperative Findings and tumor resection

Following opening of the dura, the lesion was approached through a limited corticotomy (~1.5 cm) in the mid-fusiform gyrus in order to avoid the lateral temporo-perisylvian language cortex. This provides a means to enter the cystic component and aspirate approximately 30 mL of xanthochromic fluid, producing immediate decompression and creating a compliant intracavitary working space for debulking of the solid tumor component. Tumor deb integration was performed using ultrasonic aspiration with a subpial microsurgical technique. Resection proceeded along the collateral sulcus toward the amygdalo-uncal region, allowing progressive removal of the solid tumor component while maintaining clear pial boundaries.

Throughout the procedure, motor-evoked potentials remained stable (~95% of baseline), indicating preservation of corticospinal tract integrity.

Venous Constraints and deliberate preservation

Special care was taken to preserve the regional venous anatomy. At the anteromedial margin of the tumor, where the inferior temporal veins converge to form the basal vein of Rosenthal, a thin layer of tumor capsule (~1 mm) was intentionally preserved in order to avoid injury to the venous junction. Posteriorly, the vein of Labbé was identified and preserved.

The final extent of tumor removal was therefore determined by anatomical and vascular constraints, particularly the proximity of critical venous structures and the skull base–adjacent margins of the resection cavity. Hemostasis was achieved using low-current bipolar coagulation and a flowable hemostatic matrix. The dura was closed in a watertight fashion using a collagen dural substitute, and the bone flap was secured with titanium plates.

Postoperative Course

**Figure 2:**
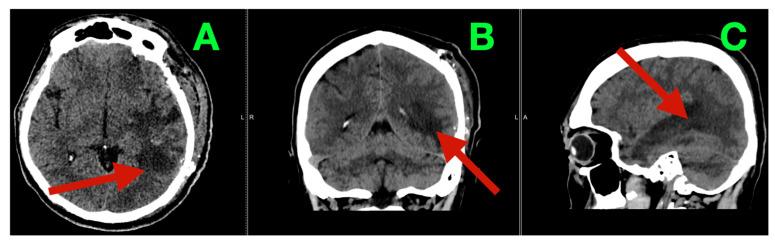
Early postoperative CT (postoperative day three). (**A**) Axial non-contrast CT demonstrating the left temporo-basal resection cavity in the former tumor bed (arrow), with expected postoperative air/fluid levels and no acute intraparenchymal hematoma or hemorrhagic cavity expansion. (**B**) Coronal reconstruction confirming effective temporal decompression (arrow), absence of clinically significant extra-axial collection, and preserved ventricular configuration without hydrocephalus. (**C**) Sagittal reconstruction outlining the skull base–adjacent inferior temporal extent of the cavity along the middle cranial fossa corridor (arrow), without radiologic features of venous congestion edema or secondary mass effect.

The patient recovered from anesthesia without new neurological deficits (Glasgow Coma Scale = 15). On postoperative day three, early postoperative CT demonstrated the anticipated left temporo-basal resection cavity with adequate decompression and no evidence of hemorrhage, hydrocephalus, or clinically relevant extrinsic collections (Figure 2A–C).

Improvement in neurological function began to occur during the first postoperative week. The patient’s NIH stroke scale decreased from 8 to 2, and his functional status improved from mRS 4 to mRS 2. Re-evaluation of his language abilities demonstrated significant improvement in naming and production of verbal output. Specifically, his Boston Naming Test scores increased from 8/30 to 26/30, and his semantic fluency improved from four to twelve words per minute. His cognitive functioning also improved to MoCA 26/30.

**Figure 3:**
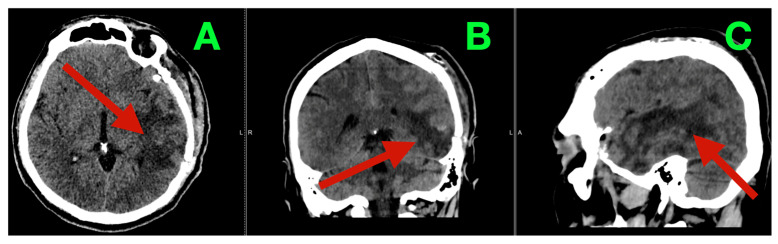
Control CT (postoperative day seven). (**A**) Axial non-contrast CT showing stable left temporo-basal postoperative cavity morphology (arrow), with resolving pneumocephalus and no delayed intraparenchymal hemorrhage. (**B**) Coronal reconstruction demonstrating maintained decompression of the temporal compartment (arrow), stable midline/ventricular anatomy, and no new extra-axial collection. (**C**) Sagittal reconstruction confirming durable skull base–parallel cavity geometry (arrow) without progressive edema, hemorrhagic conversion, or hydrocephalus.

On postoperative day seven, follow-up CT demonstrated a stable postoperative cavity without late hemorrhage or hydrocephalus (Figure 3A–C).

Histopathological evaluation of the specimen confirmed that the tumor was a high-cellularity glial neoplasm with pseudopalisading necrosis and microvascular proliferative changes indicative of glioblastoma, WHO CNS Grade IV. The molecular characterization of this tumor included the following: an IDH wild-type tumor; MGMT unmethylated status; EGFR amplified tumor; and a TERT promoter mutated tumor. These results are important for current classifications, as well as for future stratification of prognosis and selection of adjuvant therapy in adult-type diffuse gliomas. Following surgery, the patient underwent progressive reduction in corticosteroids, initiation of seizure prophylaxis using levetiracetam (500 mg BID), and referral for additional management by a neuro-oncologist.

Follow-Up

At six months after surgery, the patient continued to demonstrate good functional recovery (mRS 1; BI 95/100; L-IADL 8/8). His language performance remained stable in his everyday interactions, and he had no recurrent seizures.

**Figure 4:**
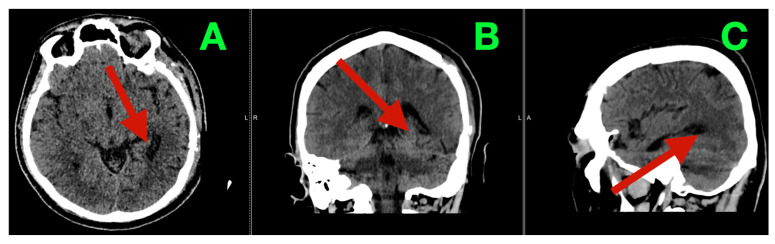
Mid-term follow-up CT (six-month control). (**A**) Axial non-contrast CT demonstrating a mature left temporo-basal postoperative cavity/encephalomalacic change (arrow) corresponding to the glioblastoma resection bed, without CT-evident nodular mass effect suggestive of large-volume recurrence. (**B**) Coronal reconstruction showing stable intracranial compartment dynamics (arrow), preserved ventricular configuration, and no hydrocephalus. (**C**) Sagittal reconstruction illustrating stable long-axis morphology of the temporo-basal cavity along the middle cranial fossa corridor (arrow), consistent with durable decompression at mid-term follow-up.

CT follow-up at six months demonstrated a mature postoperative cavity in the left temporo-basal region without evidence of hydrocephalus or large-volume recurrence (Figure 4A–C). MRI remains the preferred modality for surveillance of glioblastoma recurrence; therefore, interpretation of long-term oncologic status based solely on CT imaging should be considered limited.

The direct relationship established in this case regarding the clinical picture, radiographic findings, and operative approach to a large dominant temporo-basal glioblastoma is relevant. The rapid progression of the aphasias associated with the solid/cystic nature of the injury to the language networks in the dominant temporal lobe are consistent with each other. The main focus of the surgery was to achieve decompression of the cystic portion of the tumor, protect venous drainage pathways from the tumor, and perform a subpial microsurgical dissection to maximize preservation of functional corridors.

It would be premature to suggest that a single case provides an adequate amount of data to develop generalizable management approaches for patients with glioblastomas; however, the subsequent postoperative course of this patient suggests that attending to venous anatomy and preserving functional corridors may assist in the preservation of neurological function, as well as maintaining sufficient reserve to support additional oncologic therapies.

The clinical course in this case illustrates the inter-relationship between the neurologic manifestations of the tumor, the particular anatomic area causing those manifestations, and the intraoperative decisions regarding the extent of the surgical attempt to remove the tumor. One of the most significant challenges in the surgical management of large glioblastomas located in the dominant hemisphere’s temporo-basal region is the fact that surgery is performed at the crossroads of three important variables: (1) functional language networks, (2) base of skull anatomical limits, and (3) the location of deep cerebral veins. The choice of operative corridor to gain access to the tumor was dependent on both the cortical point of entry into the brain and the basal temporal venous anatomy. In this instance, the basal temporal venous system served as a “natural boundary” or “limit” to the maximum extent of tumor removal that could be achieved in a safe manner with surgery; therefore, care had to be taken to preserve the extremely thin capsular margin around each of the critical venous junctions. This case example illustrates how, in selected cases of glioblastoma in the dominant hemisphere’s temporo-basal region, the venous anatomy can serve as the primary limiting factor in defining the surgical boundaries rather than being merely one of many factors considered. Additionally, the longitudinal assessment of the patient’s language abilities in a systematic and structured fashion provided additional context for examining the association among the patient’s clinical manifestations, the anatomic areas demonstrable on imaging studies, and the constraints placed upon the surgeon in maximizing the degree of postoperative neurological recovery in patients who have undergone resection of glioblastomas within the dominant hemisphere. Although a single case study does not provide sufficient data to support the development of general guidelines for the treatment of glioblastoma, it can contribute to the surgeon’s decision-making process in managing tumors that occur at the juncture of multiple eloquent neural systems and complex venous anatomy.

## 3. Discussion

Dominant temporo-basal glioblastoma is a very difficult-to-treat type of brain cancer because of its tendency to grow aggressively and the eloquence of the language processing networks and the complexity of the skull base venous system in which it resides [11]. This patient’s presentation demonstrates how quickly patients with glioblastoma can develop rapidly progressive language deterioration if dominant temporal language networks are disrupted, and how thoughtful surgical planning can result in meaningful functional recovery.

Increasingly, the literature supports the concept that many neurological deficits associated with glioblastoma do not only occur from local tissue damage but also from disruption of distributed neural networks. Local tissue damage occurs through infiltration of tumor cells into the brain, vasogenic edema resulting from increased permeability of the blood–brain barrier, seizures resulting from irritation of the cortex by tumor cells and/or vasogenic edema, and alteration in the peritumoral environment leading to impaired interaction between cortical language centers and their associated white matter tracts [12]. Additionally, experimental studies demonstrate that glioma cells interact with neurons both bidirectionally and alter neuronal excitability and synaptic signaling, whereas tumor-associated microglia and macrophages contribute to extracellular matrix remodeling and progressive invasion. Therefore, in the setting of dominant temporal lesions, disruption of temporo-perisylvian language circuitry and resultant aphasic syndromes can occur. In the current case, the combination of a cystic-solid temporo-basal lesion, significant peritumoral signal abnormalities, and rapidly progressive aphasia was anatomically consistent with disruption of these language networks [13]. The postoperative improvement in the patient’s naming and language output indicates that at least some portion of the deficit likely resulted from reversible network dysfunction secondary to mass effect and edema, rather than irreversible cortical damage.

Another important point illustrated by this case is the role of venous anatomy in establishing the limits of safe resection in basal temporal surgery. Unlike most cortical regions, where the limits of safe resection are defined by the extent of cortical involvement, in the basal temporal region the limits of safe resection are typically defined by preservation of venous drainage rather than cortical boundaries. The inferior temporal veins, the uncal veins, the basal vein of Rosenthal, and the vein of Labbé form the major venous drainage pathway of the temporal lobe. Damage to these venous structures can lead to delayed venous congestion, hemorrhagic venous infarction, and progressive edema, all of which can lead to significant neurological decline despite a seemingly satisfactory immediate postoperative examination [14]. As a result, several investigators have emphasized that the extent of safe resection in this area will always be limited by venous anatomy and tract vulnerability [15]. Important recent studies (Table 1) support the importance of multiple neurobiological, vascular, and therapeutic factors in understanding the variability of phenotypic expression in dominant temporal glioblastoma and in developing effective function-preserving surgical approaches. In the current case, venous preservation was considered one of the primary limiting factors during surgical intervention, and a thin layer of tumor capsule was intentionally left near the basal vein of Rosenthal to avoid damaging the vein.

This case also demonstrates the utility of decompressive-first strategies in managing cystic or mixed cystic-solid tumors. Immediate aspiration of the cystic component provided rapid volumetric decompression, allowing intra-cystic tumor debulking without significant cortical manipulation and thereby reducing tension on the surrounding venous structures. Utilizing a combination of subpial microsurgery and continuous neurophysiological monitoring facilitated tumor removal while maintaining the physiologic integrity of corticospinal pathways and regional venous drainage.

The molecular characterization of glioblastoma continues to play an increasing role in the development of new therapeutic approaches and the clinical staging of these neoplasms. The patient described had an IDH-wildtype status, MGMT promoter unmethylated status, EGFR amplified status, and a TERT promoter mutated status; characteristics commonly found in aggressive adult-type diffuse gliomas and included within molecular classifications systems. While the focus of the current manuscript is primarily on surgical management, the molecular characterization of this neoplasm is important for assessing prognosis and selecting adjuvant therapies [24,25].

Preservation of function in patients with glioblastoma is clinically important because it allows patients to continue participating in oncologic care, including postoperative radiochemotherapy. Preservation of language function, independent living, and seizure control are all important indicators of a patient’s ability to receive subsequent oncologic treatments. In the current case, the significant improvement in the patient’s neurological function after surgery enabled the patient to be promptly referred for additional neuro-oncologic treatments [26,27].

There are limitations to the interpretation of this case. The report describes a single patient and thus cannot demonstrate the superiority of any particular operative strategy. Awake language mapping was not utilized during surgery, which is a significant limitation in the management of a dominant hemisphere lesion.

In spite of the above-mentioned limitations, this case provides a direct clinical–radiologic–surgical correlation demonstrating how rapidly progressive aphasia can develop in a patient with dominant temporo-basal glioblastoma, and how carefully planned attention to venous anatomy, decompression strategies, and functional pathways can enable successful tumor removal while preserving neurological function. The findings of this case may help guide future research and clinical practice aimed at improving function-preserving surgical techniques for tumors located in eloquent cerebral regions.

## 4. Conclusions

The purpose of this technical illustration of the primary (dominant) temporo-basal glioblastoma, was to illustrate how a successful plan for decompression of a cystic component of the tumor, through subpial microsurgical dissection while minimizing injury to larger veins, can decrease the size of the tumor and decrease the risk of secondary venous injury/disruption.

Postoperatively, the patient showed evidence of positive short-term neurologic recovery, as evidenced by improved language function and greater functional independence. However, it cannot be determined if preservation of venous drainage and/or the surrounding white matter tracts of the dominant temporal lobe were responsible for the observed improvement in neurologic function. This case study provide some evidence that preservation of venous drainage and surrounding white matter tracts may assist in maintaining the functional capabilities of the dominant temporal lobe.

This report should be viewed as a hypothesis-generating case report. It is unclear what benefits a surgeon would see from employing a venous-preserving strategy versus an alternative strategy, whether surgeons could replicate these strategies, and the long-term oncologic outcomes for patients treated with these strategies. These issues need to be resolved through comparative studies examining these strategies among a larger number of patients.

Documenting the clinical, radiologic, and surgical aspects of this case study may allow us to better understand the relationship between venous anatomy and functional networks of the brain, the relationship between the biologic characteristics of tumors and the operative approach employed, and the impact of using function-preserving surgical strategies to treat glioblastomas located within regions of eloquence to facilitate both neurologic recovery and potential future oncologic therapy.

## Figures and Tables

**Table 1:** Important research informing interpretation and management of dominant temporo-basal glioblastoma, emphasizing (i) tumor–network mechanisms relevant to rapid language dysfunction, (ii) venous/tract constraints governing maximal safe resection, and (iii) adjuvant and emerging therapy platforms where postoperative functional reserve influences treatment continuity.

References	Domain	Core Message	Case Relevance
[16]	Tumor–network biology	Neuron–glioma coupling is measurable; circuit activity can shape tumor behavior.	Supports interpreting progressive aphasia as more than a mass effect alone.
[17]	Neural-like tumor states	IDH-wildtype high-grade glioma may express synapse-related neural programs linked to adverse behavior.	Provides biologic plausibility for rapid language network deterioration.
[18]	Tumor–brain heterogeneity	Tumor–neuron interaction patterns vary across patients and subtypes.	Supports cautious, non-universal interpretation of network vulnerability in this case.
[19]	Venous surgical risk	Injury to cortical/deep venous drainage may cause delayed congestion or hemorrhagic venous infarction.	Justifies venous-preserving resection and intentional capsular preservation at unsafe venous interfaces.
[20]	Treatment continuity	Real-world outcomes are influenced by the feasibility and adherence to postoperative therapy.	Links functional preservation to the ability to proceed with adjuvant care.
[21]	Drug-delivery innovation	BBB-opening strategies may expand therapeutic access in high-grade glioma.	Preserving function may help maintain eligibility for evolving treatment platforms.
[22]	Salvage local therapy	SRS may have a role in selected recurrences, with context-dependent toxicity.	Supports a restrained discussion of focal salvage options, not frontline treatment.
[23]	Targeted therapy limits	Emerging targeted platforms show promise but remain constrained by resistance biology.	Reinforces the value of molecular profiling while avoiding therapeutic overstatement.

## Data Availability

The data presented in this study are available on request from the corresponding author.
